# COVID-19 Infection among Patients Presenting to a Tertiary Care Centre: A Descriptive Cross-sectional Study

**DOI:** 10.31729/jnma.7932

**Published:** 2023-01-31

**Authors:** Meera Bista, Raunak Bista, Vabesh Mishra, Inku Basnet

**Affiliations:** 1 Department of Otorhinolaryngology, Kathmandu Medical College and Teaching Hospital, Sinamangal, Kathmandu, Nepal; 2 Kathmandu Medical College and Teaching Hospital, Sinamangal, Kathmandu, Nepal

**Keywords:** *blood group*, *COVID-19*, *pandemic*

## Abstract

**Introduction:**

Coronavirus disease-19 infection is caused by the coronavirus and has taken a toll throughout the world. The aim of this study was to find the prevalence of coronavirus disease-19 infection among patients presenting to a tertiary care centre.

**Methods:**

A descriptive cross-sectional study was conducted at the fever clinic of a tertiary care centre between January 2021 to September 2021 after receiving ethical approval from the Institutional Review Committee (Reference number: 2011202001). Convenience sampling was done. Data were collected from the records of patients diagnosed with real-time polymerase chain reaction) test in the sample group. Point estimate and 95% Confidence Interval were calculated.

**Results:**

Among 230 patients presenting to the fever clinic, 130 (56.52%) (50.11-62.93, 95% Confidence Interval) were diagnosed with coronavirus disease-19.

**Conclusions:**

Our study found that the prevalence of coronavirus disease-19 was higher when compared to similar studies conducted in similar settings.

## INTRODUCTION

The novel coronavirus disease (COVID-19) pandemic has taken its toll on the whole world. The disease's vast influence on our daily life patterns has raised huge concerns about the different parameters associated with it. A study regarding blood group prevalence in the disease quotes the prevalence of blood group A as the most susceptible and group O as the least susceptible.^1-3^

COVID-19 infection could lead to a critical condition, and the susceptibility of the COVID-19 virus might have an association with blood group type rendering it more vulnerable to the disease.^4^ However, studies on the prevalence of the infection and the blood group types are not adequate in our setting.

Hence, the aim of this study was to find the prevalence of COVID-19 infection among patients presenting to a tertiary care centre.

## METHODS

A descriptive cross-sectional study was conducted at Kathmandu Medical College and Teaching Hospital among patients presenting to the fever clinic between January 2021 to September 2021 after receiving ethical approval from the Institutional Review Committee (Reference number: 2011202001). All the patients presenting during the study period to the fever clinic were included and the patients who didn't provide consent were excluded. Convenience sampling was done and the sample size was calculated using the formula:


$$\begin{array}{ll} \text{n} & ={\text{Z}}^{2}\times \frac{\text{p}\times \text{q}}{{\text{e}}^{\text{2}}}\\ & =1.96^{2}\times \frac{0.50\times 0.50}{0.05^{2}}\\ & =194 \end{array}$$

Where,

n = minimum required sample size
Z = 1.96 at 95% Confidence Interval (CI)
p = prevalence of COVID-19 infection, 14.8%^5^
q = 1-p
e = margin of error, 5%

Hence, the minimum required sample size was 194 for the study. After adding 10% to address missing data, a sample size of 216 was obtained. However, a sample of 230 patients was taken for the study.

Data were collected from the records of patients diagnosed with COVID-19 infection using the RT-PCR (real-time polymerase chain reaction) test. Data regarding age, gender, sociodemographic details and ABO grouping were also collected. ABO grouping was done by using the standard slide method, using Anti-A, Anti-B and Anti-D taken on glass slides were used. Point estimate and 95% Confidence Interval were calculated.

## RESULTS

Among 230 patients presenting to the fever clinic, 130 (56.52%) (50.11-62.93, 95% Confidence Interval) were diagnosed with COVID-19 infection. The mean age of these patients was 41.98±18.03 years ranging between 3 to 84 years of age. Out of them, 70 (36.92%) were males and 60 (40%) were females.

**Figure 1:**
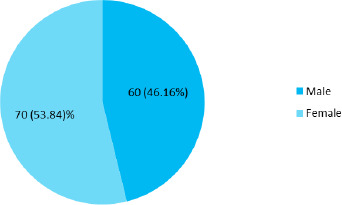
Gender-wise distribution of the patients with COVID-19 infection (n= 130).

Mortality was seen in 30 (23.07%) of the patients who had a mean age of 57.17±13.95 years. Out of these 30 patients, 22 (73.33%) were males and eight (26.67%) were females. The most common blood group in COVID-19-positive patients was blood group O in 45 (34.61%) followed by blood groups A in 31 (23.84%), B in 28 (21.53%) and AB in 26 (20%) patients. Among the COVID-19 patients with mortality, blood groups O and A were seen in 18 (60%) patients (Table 1).

**Table 1:** Blood groups of patients who died of COVID-19 infection (n= 30).

Blood groups	n (%)
A	9 (30)
B	7 (23.33)
AB	5 (16.67)
O	9 (30)

## DISCUSSION

Our study found that the prevalence of COVID-19 infection was 56.52% in our setting and was more common among males. Studies in Nepal have reported the prevalence of COVID-19 infection to be 14.8%, 7.05% and 2.25%.^5-7^

There are certain diseases which have been proven to have certain relations with blood groups. Microangiopathic hemolytic anaemia has a strong relation with Non-O blood groups, making them a risk factor for this group of disease.^8^ Venous thromboembolism and ischemic stroke are seen in blood groups A and AB.^9^ In another study, showed that the von Willebrand factor plays a major role in the clotting mechanism so a higher risk of deep vein thrombosis in individuals belonging to the blood group Non-O is seen due to a higher level of this factor.^10^ Non-A group is also associated with a high risk of Cerebrovascular accidents.^11^ Blood group AB has been found to have a significant association with preeclampsia with an increased risk of 2.1 folds.^12^ Protection against falciparum malaria by reduction of rosette formation is seen in blood group O.^13^ Some studies suggest an association of blood group with malignancies. A positive correlation has been seen with blood group B and ovarian cancer^14^ and blood group A with chronic hepatitis infection and pancreatic cancer.^15^ There is a relation between peptic ulcers, gastric cancers and type of blood group.

Such relation is sought in COVID condition too. This study shows infection to be more common in blood group O followed by A, B and AB. A similar result was shown by a study published in Ann Hematol in 2020.^16^ But most of the studies show group O to be less and A to be most susceptible to the disease.^1,17,18^

Our study was a descriptive cross-sectional study which limited its ability to make an association between the study variables. Also, the small sample size and single-centric nature of the study limit the generalizability of the findings of the study. Hence, a study with a higher study design and a larger sample size is warranted.

## CONCLUSIONS

Our study found that the prevalence of COVID-19 was higher when compared to similar studies conducted in similar settings. There are certain diseases which have been proven to have certain relations with blood groups similar to COVID-19 that also show relation is sought in COVID-19 conditions further a study with a higher study design and a larger sample size is warranted.
